# On the local warming potential of urban rooftop photovoltaic solar panels in cities

**DOI:** 10.1038/s41598-023-40280-9

**Published:** 2023-09-20

**Authors:** Ansar Khan, Mattheos Santamouris

**Affiliations:** 1 Department of Geography, Lalbaba College, University of Calcutta, Kolkata, India; 2 School of Built Environment, University of New South Wales, Sydney, Australia

**Keywords:** Climate sciences, Environmental sciences, Environmental social sciences, Energy science and technology, Engineering

## Abstract

Understanding and evaluating the implications of photovoltaic solar panels (PVSPs) deployment on urban settings, as well as the pessimistic effects of densely populated areas on PVSPs efficiency, is becoming incredibly valuable. Thus, the deployment of low-efficiency, low-cost, and widely available PVSPs may diminish total solar reflectance, raising the risks of PVSPs-based urban heating, particularly during the summertime heatwaves. This study employs and assesses physical parameterizations that account for the impact of PVSPs on Sydney’s urban environment in the context of the mesoscale model weather research and forecasting (WRF). To account for the impacts of PVSPs, the parameterization presented in this paper assumes that PVSP arrays are parallel, detachable from roofs, and consist of a single layer. Results showed that increasing PVSPs can raise peak summer ambient temperatures by up to 1.4 °C and surface temperatures by up to 2.3°C at city-scale. Temperature variability was found between the city’s eastern and western parts due to the presence of PVSPs. In addition, local warming effects of PVSP were observed at urban district-scale as well. The large-scale deployment of PVSPs at local district-scale of the Sydney during a typical hot day caused air temperature to rise by 1.5 °C during the daytime and decrease by 2.7 °C at nighttime. The patterns of the city’s ambient temperature distribution were found to be strongly dependent on synoptic meteorological conditions and advection flow strength. The maximum increases in sensible heat flux and latent heat flux were 245.5 Wm^−2^ and 11.5 Wm^−2^, respectively. Wind speed may be raised by up to 1.2 ms^−2 ^due to regional low effect over city domain. As a result, large-scale deployment of PVSPs promotes advective flow between the city and its environs. Modification of the PVSPs in Sydney results in an increase in planetary boundary layer (PBL) heights of up to 537.9 m above the city and may lower pollutant concentrations at ground level. The advent of sea breeze in the city’s eastern parts, which reduces the temperature of the coastal zone, along with inland westerly winds, which heat the city’s western zones, lessened the intensity of the urban heat island (UHI) phenomenon induced by PVSPs warming. The findings of this study can be used to help policymakers make informed decisions about the use of PVSPs systems. PVSPs with a high solar reflectance in wavelengths that do not convert solar energy to electricity can be considered as an alternative solution to reduce local warming in urban environments.

## Introduction

The recent and anticipated future expansion of photovoltaic solar panel (PVSPs) in urban environments is exciting from the aspect of renewable energy generation, but it also poses serious challenges. In terms of ambient temperature, wavelength-dependent radiation flux, shading of panels by neighboring structures, and shadow cast by panels on occupants underneath, PVSPs both influence and are affected by their local surroundings^1^. While the performance of PVSPs renewable energy has risen, there are still doubts over whether PVSPs have a ‘heat island’ effect, comparable to how an increase in ambient temperatures in cities generates an urban heat island effect (UHIE). The shadowing effects produced by nearby buildings or an increase in air temperature due to the presence of PVSPs. Like the UHIE, large scale PVSPs cause a landscape change that impacts albedo, making the changed roof darker and hence less reflective^2^. A recent study on the environmental impact of solar panels revealed that they may significantly warm cities throughout the day. According to the research, this heating can also affect the performance of PVSPs^1^.

The ‘effective albedo’ approach has significantly aided in the study of the relationship between PVSP modules and urban air temperature, however it is a simplification that can lead to inaccurate estimations of the PVSPs energy balance^3–8^. Salamanca et al. (2016)^9^ parameterized and implemented the PVSPs scheme proposed by Masson et al. (2014)^10^ in weather research and forecasting (WRF)/building effect parameterization (BEP) + building energy model (BEM). The temperature of the PVSPs, however, is not explicitly computed. Because of the elevated gap between the roof surface and the PVSPs, heat is convected away from the PVSPs on both the top and bottom surfaces. Local wind speeds and thus convection coefficients may be slightly higher on the top surface. As a result, these findings require serious assessment, particularly in light of the implications for research strategy and policy. Masson et al. (2014)^10^ shown that PVSP arrays may lower near-surface air temperature, particularly at night. Finally, Salamanca et al. (2016)^9^ investigated a novel PVSP parameterization in conjunction with the multilayer urban canopy (MLUC) schemes such as BEP, and BEM for thermal management of cities^11,12^ for the cities of Phoenix and Tucson, detecting a reduction in both near-surface temperature and energy demand for air conditioning systems (ACSs) due to effective albedo approach. The PVSP reduces thermal comfort somewhat on average at city scale. This finding contradicts the findings of Masson et al. (2014)^10^ and Salamanca et al. (2016)^9^, but it is consistent with previous observational data indicating PVSP often boost temperature throughout the day^1,2,13^. The previous results were disillusioned that most of studies introduced inaccurate representations of the energy balance of PVSPs in the urban environment and, as a result, made claims about the potential for PVSPs, for example, to cool the urban environment when, in fact, the energy balance is much more complex, and the implications for the urban environment are correspondingly complex. When convective flux from the bottom surface is ignored, the estimated convective heat flux to the environment is underestimated. One has to do with PVSP and their relationship to urban energy balance. It is also found that those PVSPs may significantly warm the urban environment during the day but typically cool it at night. Another key result was that, for a variety of reasons, PVSPs do not perform as well in urban contexts as they do in other settings, such as rural and suburban regions^1^.

Many studies have examined PVSPs from a modeling standpoint, while others have examined actual or empirical data. A study in PVSP arrays in the desert, as well as in a comparable desert environment not far away from the reference site. It was determined that the average air temperature at 1.5 m at the PVSP array site was around 1.3 °C higher than the reference site, which was a non-PVSP site, in that observational study^13^. At night, Broadbent et al. (2019)^13^ also found that there was little influence from the PVSP array on air temperature. As a result, observational study led to the conclusion that PVSPs do have this warming effect during the day, but the effect may be extremely weak or nonexistent at night, making it impossible to measure. Other studies, particularly modeling ones, have already shown that PVSPs might offer daytime cooling. They did, however, depict the PVSP panels incorrectly, neglecting the fact that PVSPs may convect heat from both the top and bottom surfaces^9,10^. The temperature coefficient of traditional silicon-based PVSPs implies that the surface temperature of the solar cells impacts their efficiency. As a result, the PVSP surface will be less efficient in warm climatic regions. Solar cell temperature normally has a temperature coefficient of 4%/°C. This implies that, while PVSPs are normally evaluated at a conventional test temperature of 25 °C, operating in an urban setting—where the PVSPs surface temperature often reaches 65–70 °C or even hotter—reduces total efficiency by 10–15%^7,14^.

Several studies have found that installing PVSPs on a building’s rooftop lowers the yearly energy consumption of the ACS^15,16^. This makes logical sense given that the PVSPs provide shade from direct sunlight. As a result, only a fraction of the solar load that would normally travel through the roof surface is received by the buildings. And that is how the city would anticipate profiting from AC. Similarly, there may be a heating penalty and an increase in seasonal heating demand associated with rooftop PVSPs in the winter where it is desired that solar radiation reach the building surface but PVSPs effectively shade the buildings^17^. It is reported that in extremely hot climates, such as Phoenix, USA, PVSPs have a complex set of trade-offs for residential building stock^8^. However, they have the advantage of quickly shielding buildings from the sun during the day. However, at night, the PVSPs panels obscure the building’s view of the sky, slowing heat loss, whereas the building roof surface would normally radiate its energy out into space, aiding in quick cooling. As a result, the necessity for AC in the residential buildings increases at night^1,2,9^.

As a result of the transition from diagnosing knowledge gaps to considering how to address those gaps, it would be advantageous to provide a more comprehensive assessment of PVSPs in the local environment. We sought to understand not just how installing PVSPs systems in the urban environment impacts buildings and urban air temperatures, but also how the urban environment influences PVSPs system performance. Therefore, it is critical to remember that on this issue is dependent on what comparing a specific application against. For example, deploying PVSPs on an existing black roof has less of an impact on the urban thermal environment than installing same PVSPs on a white roof, because a white roof is often a relatively cool surface. PVSPs put on a white roof typically capture 90% of the solar energy^18^. While PVSPs do convert some of the energy, the average panel today is only approximately 16–20% efficient. These panels absorb a large amount of energy from the sun, converting some of it into electricity but then heating up since they cannot utilize all of it. As a result, PVSPs emit a lot of heat into the environment. They are often placed at an elevation, such as above the surface of a roof or above ground level in a field. As a consequence of having two heated surfaces, one on top and one on bottom of the panels. As a result, when air travels over these panels, it picks up heat roughly twice as effectively as it would on a similar-temperature building or ground surface^8,18^.

Based on the available studies, it is clear that the two-sided heat exchange from PVSPs is resulting in increased heat being added to the urban environment. The WRF model is now able to parameterize the surface in such a way to capture this approximately doubling of the heat exchange at building or city scale^19^. Therefore, an improved comprehension of the physical mechanisms behind the changes induced by PVSPs is necessary in order to quantify their effects on the urban environment, for a wide range of urban buildings, and under various climatic conditions. Several studies have been performed to quantify the impact of PVSPs at the building scale, using either field campaigns or numerical simulations^1,2,17^. However, because the impact of PVSPs varies depending on urban geometry, thermal characteristics of building materials, and climatic conditions, the results cannot be easily up scaled to analyze thermal impacts at the city scale; an alternate approach is required. In addition, PVSPs confront specific obstacles in urban environments, such as limited usable rooftop area, shade from tall buildings, and increased air pollution. The influence of densely inhabited areas on the efficiency of PVSPs must be investigated, as this has substantial consequences for their widespread application in cities. To this purpose, we used mesoscale meteorological model to study the city-wide influence of PVSPs, using urban parameterizations with varying levels of complexity.

In the framework of the mesoscale model WRF, this study uses and evaluates physical parameterizations that account for the effect of PVSPs on the urban environment of Sydney. The two research questions are placed in the context of PVSPs performance (a) How do urban environmental factors influence PVSPs performance when PVSPs are placed in cities, (b) What are the thermal impacts of PVSPs on the urban environment? We address these issues by designing different fraction of PVSPs on rooftop and their numerical evaluation at city scale. The unique application uses numerical simulations in real urban settings for typical heatwave conditions to assess the sensitivity of near-surface standard meteorological field and urban climate impact to varied PVSPs fractions.

## Results

The results of the control scenario (cool roofs) are used as a reference to compare with four solar PVSPs scenarios. The predictions of the mesoscale model with conventional roofs have been compared against the collected data from the main ground climatic stations in Sydney to ensure the robustness and accuracy of the model. Our previous study^20^ assessed the impact of PVSPs on urban temperature and corresponding cooling load penalties in 17 representative building types in Sydney during the two summer months of January and February, using standard roofs (e.g., roof albedo = 0.2) as a reference scenario. According to the study, when 25–100% of roof areas are covered by PVSPs, the ambient air temperature may rise by 0.6–2.3 °C. To avoid repeating results, we used cool roofs as a reference scenario for analyzing the performance of PVSPs (rather than validating the model) in this study for two reasons: (a) the cool roofs reflect more solar radiation back towards the panels, and (b) it lowers the ambient temperature, which increases the performance of the PVSPs modules. The results of the control scenario with cool roofs are presented for two months of summer. The simulated summer period is from 31 December 00:00 h, 2016 to 1 March 00:00 h, 2017. The PVSPs scenario presented here have been analyzed during the summer period for 59 days of two months (January and February). These two months were warmer than average during 2017 for both daytime and overnight temperatures in Greater Sydney. The mean temperature at Observatory Hill was the second-warmest on record, behind 2016^21^.

### Ambient temperature

**Figure 1:**
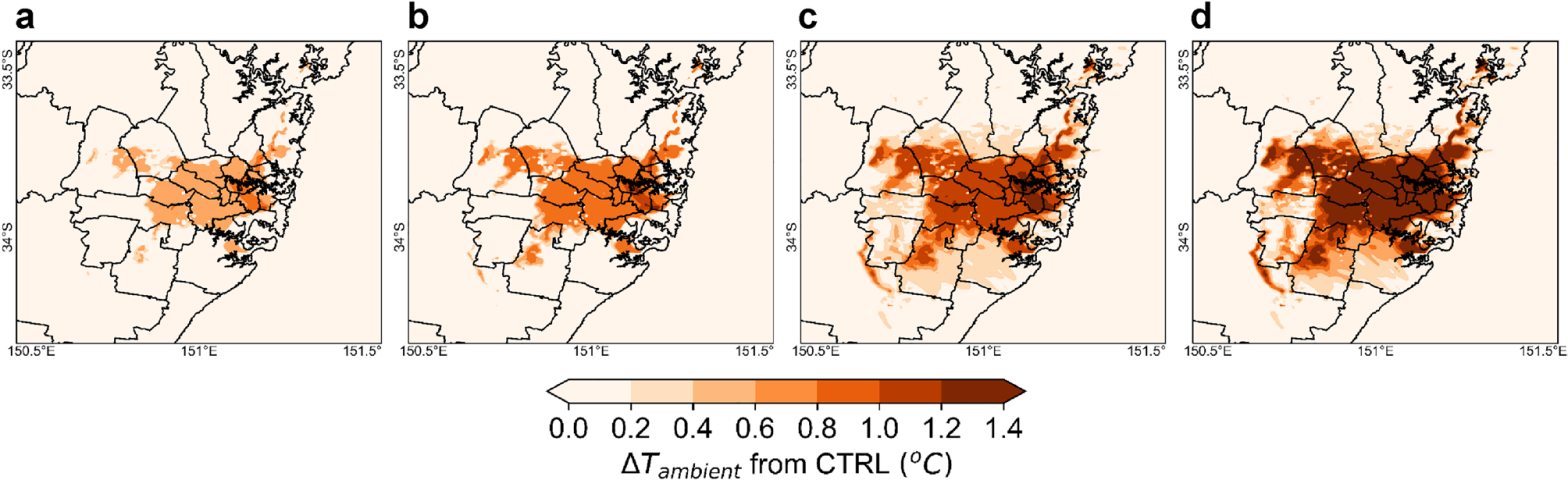
Increase of ambient temperature at peak hour (14:00 LT) for (**a**) PVSPs 25%, (**b**) PVSPs 50%, (**c**) PVSPs 75%, and (**d**) PVSPs 100%. This map has been created from building rooftop with PVSP scenarios minus cool roofs without building rooftop PVSPs for the same urban grid cell. This map was prepared by the first author with the help of WRF-Python (https://github.com/NCAR/wrf-python) and does not require any permission from anywhere.

Ambient temperatures are influenced by the surface energy balance equation in the WRF/BEP + BEM urban modeling system^22^. Under the PVSPs scenario, the ambient temperature at 14:00 LT ranges between 21.1 and 42.2 °C for PVSPs 25%, 21.6–42.9 °C for PVSPs 50%, 21.9–43.5 °C for PVSPs 75%, and 22.5–43.8 °C for PVSPs 100% scenarios. At 06:00 LT, it varies from 18.6 to 33.7 °C for PVSPs 25%, 18.8–33.9 °C for PVSPs 50%, 19.3–34.2 °C for PVSPs 75%, and 19.6–34.5 °C for PVSPs 100%.The results show that with the implementation of the PVSP on urban roof surface, the maximum increase in the peak ambient temperature (T_ambient_) is 0.5 °C, 0.7 °C, 1 °C, and 1.4 °C for PVSPs 25%, PVSPs 50% PVSPs 75% and PVSPs 100%, respectively over central business district (CBD) and eastern Sydney compared to control scenario. The average ambient temperature increases at 14:00 LT over the whole summer are 0.4 °C for PVSPs 25%, 0.6 °C for PVSPs 50%, 0.9 °C for PVSPs 75% and 1.1 °C for PVSPs 100%. But, during 18:00 LT, the maximum decrease of the ambient temperature is − 0.2 °C, − 0.4 °C, − 0.6 °C, and − 0.7 °C for PVSPs 25%, PVSPs 50% PVSPs 75%, and PVSPs 100%, respectively over eastern Sydney and the average decrease of summer months varies between − 0.1 and − 0.5 °C (Fig. 1). This is because urban areas are often warmer than rural regions—a phenomenon known as the UHI effects. The installation of PVSPs in urban environments may have an additional detrimental influence on PVSP efficiency and overall power generation. The amplitude of the UHI in Sydney is generally largest at night, but it can reach 6 °C or greater during the day, contributing to higher PVSP surface temperatures due to convective flow between roof and panel surface.

### Surface temperature

**Figure 2:**
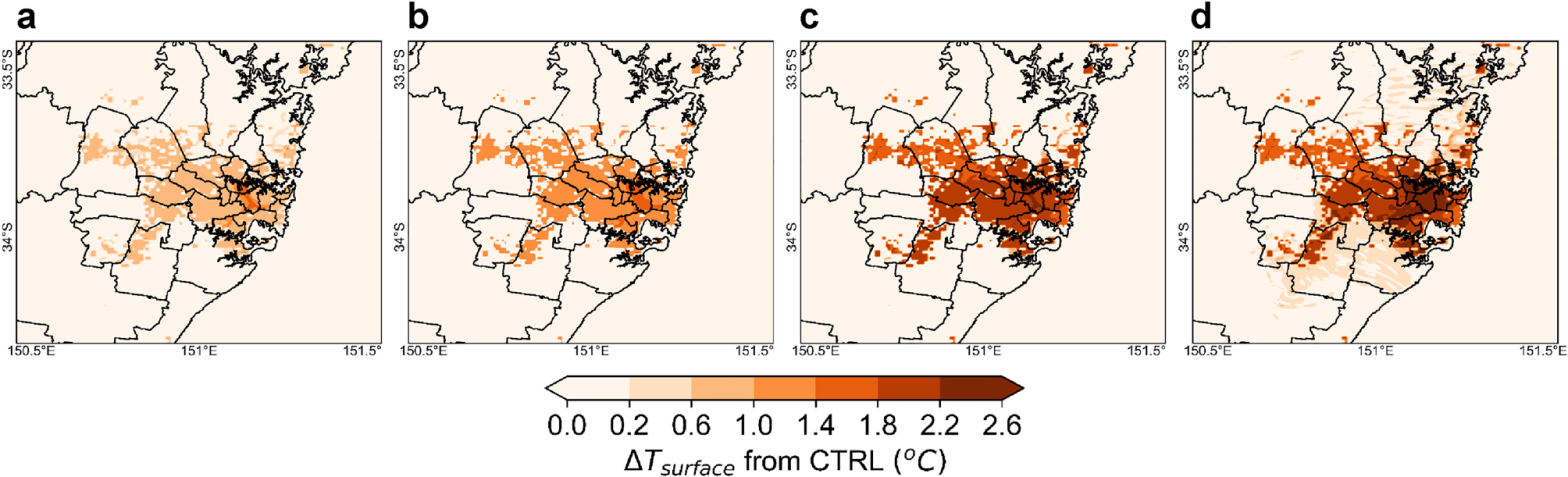
Increase of surface temperature at peak hour (14:00 LT) for (**a**) PVSPs 25%, (**b**) PVSPs 50%, (**c**) PVSPs 75%, and (**d**) PVSPs 100%. This map has been created from building rooftop with PVSP scenarios minus cool roofs without building rooftop PVSPs for the same urban grid cell. This map was prepared by the first author with the help of WRF-Python (https://github.com/NCAR/wrf-python) and does not require any permission from anywhere.

The urban surface temperature is a weighted area average of the roof and road surface temperatures. Once PVSPs are installed, the roof temperature is calculated as a weighted area average considering both the fraction with and without PVSPs with the corresponding surface energy balance modified due to their presence. Under the PVSPs scenario, the surface temperature (T_surface_) during 14:00 LT ranges between 25.9 and 49.2 °C for PVSPs 25%, 26.2–49.7 °C for PVSPs 50%, 26.9–50.3 °C for PVSPs 75% and 27.4–50.8 °C for PVSPs 100% over city. At 6:00 LT, it varies from 21.3 to 37.2 °C, 21.6–37.5 °C, 21.9–33.9 °C and 22.1–38.1 °C for PVSPs 25%, PVSPs 50%, PVSPs 75% and PVSPs 100%, respectively. The maximum increase of surface temperature during 14:00 LT is 0.7 °C for PVSPs 25%, 1.2 °C for PVSPs 50%, 1.8 °C for PVSPs 75%, and 2.3 °C for PVSPs 100% over eastern Sydney and Southern Sydney. The average increase of urban surface temperature during 14:00 LT of summer months is 0.6 °C, 0.9 °C, 1.6 °C, 2.1 °C for PVSPs 25%, PVSPs 50%, PVSPs 75%, and PVSPs 100%, respectively over the urban domain. Using the PVSPs on the urban roof surface, the maximum decrease of surface temperature during 18:00 LT is − 0.3 °C for PVSPs 25%, − 0.7 °C for PVSPs 50%, − 0.9 °C for PVSPs 75%, and − 1.1 °C for PVSPs 100% scenario. The average decrease of urban surface temperature during 18:00 LT of summer months are − 0.2 °C, − 0.5 °C, − 0.7 °C and − 0.9 °C for PVSPs 25%, PVSPs 50%, PVSPs 75% and PVSPs 100% scenario, respectively (Fig. 2).

### Sensible heat flux

**Figure 3:**
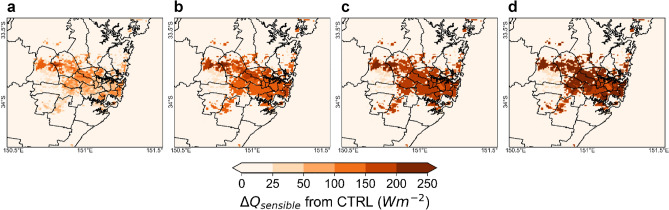
Increase of sensible heat flux at peak hour (14:00 LT) for (**a**) PVSPs 25%, (**b**) PVSPs 50%, (**c**) PVSPs 75%, and (**d**) PVSPs 100%. This map has been created from building rooftop with PVSP scenarios minus cool roofs without building rooftop PVSPs for the same urban grid cell. This map was prepared by the first author with the help of WRF-Python (https://github.com/NCAR/wrf-python) and does not require any permission from anywhere.

In general, cool roofs have a lower sensible heat flux than conventional roofs (dark surfaces). When compared to dark roofs, cool roofs can reduce sensible heat by reflecting more solar radiation back towards the panels, lowering the ambient temperature and so increase the performance of the PVSPs modules. In other words, PVSPs directly increase sensible heat shown due to PVSPs generating convective heat during operation, and secondary effects such as shade and airflow alterations might have an impact on the local sensible heat transfer dynamics. The WRF/BEP + BEM reasonably computed the sensible heat flux from the urban surface. The maximum and average sensible heat flux (Q_sensible_) over city during 14:00 LT are 175.3 Wm^−2^ and 110.3 Wm^−2^ for PVSPs 25%, 236.4 Wm^−2^ and 185.6 Wm^−2^ for PVSPs 50%, 297.5 Wm^−2^ and 265.3 Wm^−2^ for PVSPs 75%, 358.9 Wm^−2^ and 298.6 Wm^−2^ for PVSPs 100% scenario, respectively. At 18:00LT, the average sensible heat flux is 45.3 Wm^−2^, 56.2 Wm^−2^, 62.1 Wm^−2^ and 76.4 Wm^−2^ for PVSPs 25%, PVSPs 50%, PVSPs 75% and PVSPs 100% scenario, respectively. The maximum increase of the sensible heat flux during 14:00 LT is 57.5 Wm^−2^ for PVSPs 25%, 110.9 Wm^−2^ for PVSPs 50%, 185.6 Wm^−2^ for PVSPs 75% and 245.5^−^Wm^−2^ for PVSPs 100% scenario, respectively over CBD and inner west. The average increase of sensible heat flux at 14:00 LT of summer are 51.2 Wm^−2^, 102.3 Wm^−2^, 176.4 Wm^−2^ and 238.5 Wm^−2^ for PVSPs 25%, PVSPs 50%, PVSPs 75% and PVSPs 100% scenario, respectively over urban domain. At 18:00LT, the maximum increase of summer month of sensible heat flux are 6.5 Wm^−2^ for PVSPs 25%, 11.8 Wm^−2^ for PVSPs 50%, 18.3 Wm^−2^ for PVSPs 75% and 26.2 Wm^−2^ for PVSPs 100% scenario. The average increase of sensible heat flux of summer during 18:00 LT are 3.4 Wm^−2^, 8.6 Wm^−2^, 13.9 Wm^−2^ and 21.2 Wm^−2^ for PVSPs 25%, PVSPs 50%, PVSPs 75% and PVSPs 100% scenario, respectively (Fig. 3).

### Latent heat flux

**Figure 4:**
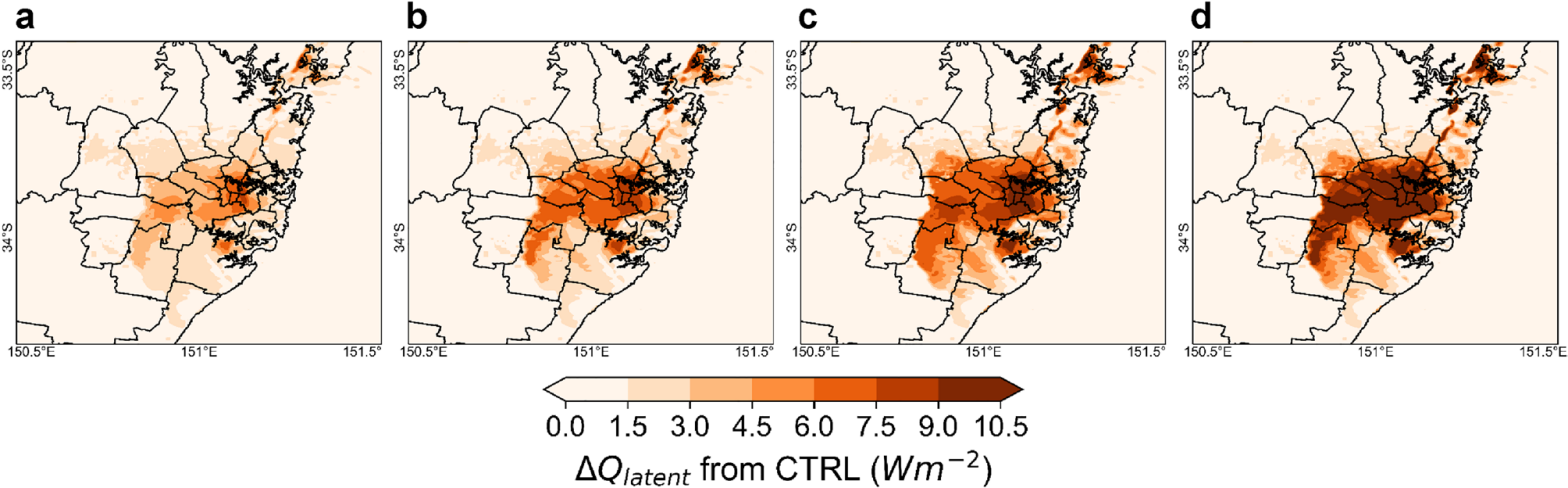
Increase of latent heat flux at peak hour (14:00 LT) for (**a**) PVSPs 25%, (**b**) PVSPs 50%, (**c**) PVSPs 75%, and (**d**) PVSPs 100%. This map has been created from building rooftop with PVSP scenarios minus cool roofs without building rooftop PVSPs for the same urban grid cell. This map was prepared by the first author with the help of WRF-Python (https://github.com/NCAR/wrf-python) and does not require any permission from anywhere.

PVSPs do not boost latent heat flux directly. PVSPs are generally put on rooftops to provide shade to the areas below. PVSPs can lower the amount of solar radiation reaching the surface by obstructing direct sunlight, which can impact the warmth of the surrounding air. When a surface is shaded, it gets less energy, resulting in a lower temperature rise and possibly less evaporation, both of which affect the latent heat flux. Additionally, PVSPs have the potential to change the micro environment surrounding their installation location. They act as a wind break and may slow air movement in shaded places. Reduced airflow can impede moisture dispersion and affect evaporation rate, affecting latent heat flux. In addition, increased wind speed also can enhance evaporation, transport moisture, promote mixing and dilution, and affect urban boundary layer (UBL) dynamics, all of which can contribute to an increase in latent heat flux. The maximum and average latent heat flux (Q_latent_) over city during 14:00 LT are 35.5 Wm^−2^ and 23.8 Wm^−2^ for PVSPs 25%, 37.5 Wm^−2^ and 25.1 Wm^−2^ for PVSPs 50%, 39.5 Wm^−2^ and 26.5 Wm^−2^ for PVSPs 75% 42.5 Wm^−2^ and 28.5 Wm^−2^ for PVSPs 100% scenario. At 18:00 LT, the average latent heat flux are 7.4 Wm^−2^, 8.3 Wm^−2^, 9.3 Wm^−2^ and 10.4 Wm^−2^ for PVSPs 25%, PVSPs 50%, PVSPs 75% and PVSPs 100% scenario, respectively. The maximum increase of the latent heat flux during 14:00 LT is 4.3 Wm^−2^ for PVSPs 25%, 6.4 Wm^−2^ for PVSPs 50%, 8.3 Wm^−2^ for PVSPs 75% and 11.5 Wm^−2^ for PVSPs 100% scenario over CBD and eastern Sydney. The average increase of latent heat flux at 14:00LT of summer months are 2.9 Wm^−2^, 4.3 Wm^−2^, 5.6 Wm^−2^ and 7.6 Wm^−2^ for PVSPs 25%, PVSPs 50%, PVSPs 75% and PVSPs 100% scenario, respectively over urban domain. At 18:00 LT, the maximum and average increase of summer month of latent heat flux are 2.7 Wm^−2^ and 1.7 Wm^−2^ for PVSPs 25%, 4.1 Wm^−2^ and 2.7 Wm^−2^ for PVSPs 50%, 5.6 Wm^−2^ and 3.6 Wm^−2^ for PVSPs 75% 7.3 Wm^−2^ and 4.7 Wm^−2^ for PVSPs 100% scenario over eastern Sydney. At, 06:00 LT, the maximum increase of latent heat flux are 1.9 Wm^−2^, 2.1 Wm^−2^, 2.9  Wm^−2^, and 4 Wm^−2^ for PVSPs 25%, PVSPs 50%, PVSPs 75% and PVSPs 100% scenario respectively over urban domain (Fig. 4).

### Surface wind

**Figure 5:**
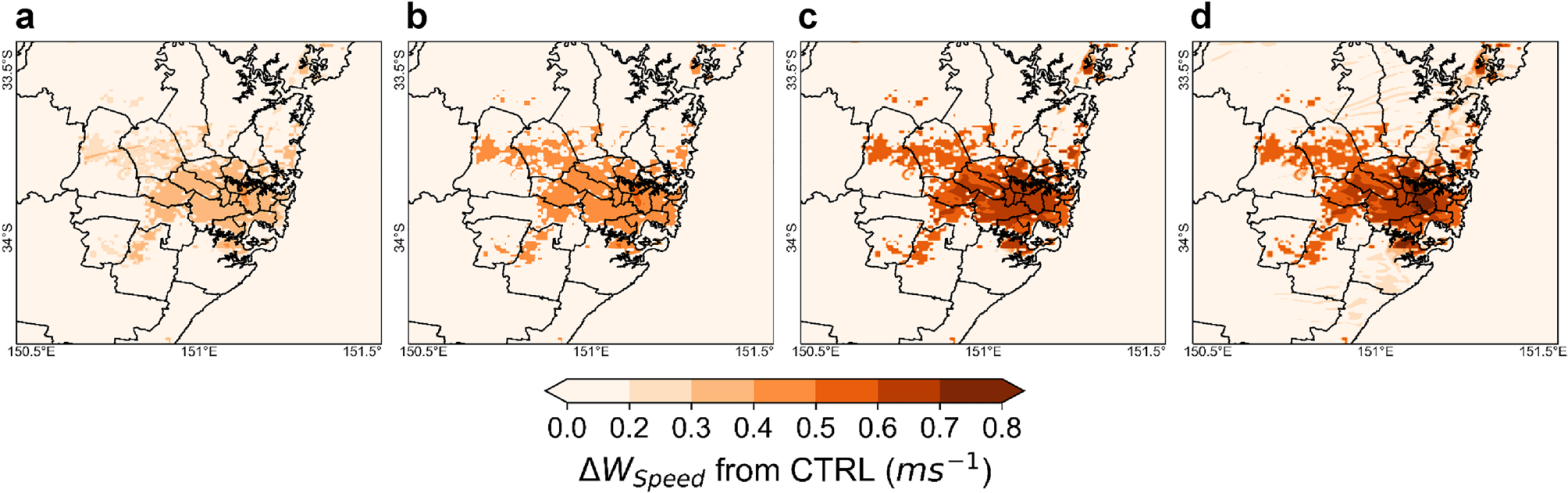
Increase of wind speed at peak hour (14:00 LT) for (**a**) PVSPs 25%, (**b**) PVSPs 50%, (**c**) PVSPs 75%, and (**d**) PVSPs 100%. This map has been created from building rooftop with PVSP scenarios minus cool roofs without building rooftop PVSPs for the same urban grid cell. This map was prepared by the first author with the help of WRF-Python (https://github.com/NCAR/wrf-python) and does not require any permission from anywhere.

PVSPs boost regional wind speed over Sydney. This is the increment for wind coming from the east (sea breeze) and west (inland). The diurnal cycle of regional wind over Sydney is impacted by a number of factors, including local topography, proximity to the coast, and prevailing weather patterns. Wind speed has a significant influence on the exchange of moisture between the surface and the atmosphere. Increased wind speed can boost evaporation, transport moisture, promote mixing and dilution, and influence UBL dynamics, all of which can contribute to an increase in latent heat flux. Under the control scenario simulation, the average wind speeds (W_speed_) are 8.8 ms^−1^, 9.4 ms^−1^ and 8.9 ms^−1^ during 06:00 LT, 14:00 LT and 18:00 LT respectively over the city. The maximum increase of wind speed compared to control case is 0.4 ms^−1^ for PVSPs 25%, 0.6 ms^−1^ for PVSPs 50%, 0.7 ms^−1^ for PVSPs 75% and 1.2 ms^−1^ for PVSPs 100% scenario during 14:00LT and 0.3 ms^−1^, 06 ms^−1^, 0.7 ms^−1^, and 0.9 ms^−1^ for PVSPs 25%, PVSPs 50%, PVSPs 75% and PVSPs 100% scenario, respectively during 18:00LT over inner west, CBD, and lower north shore, western parts of CBD. The average increase of wind speed for whole summer months during 14:00LT is 0.2 ms^−1^, 0.4 ms^−1^, 0.5 ms^−1^ and 0.7 ms^−1^ for PVSPs 25%, PVSPs 50%, PVSPs 75% and PVSPs 100% scenario, respectively over the city (Fig. 5). At 6:00LT, the average increase of wind speed of summer months is 0.1 ms^−1^ for PVSPs 25%, 0.2 ms^−1^ for PVSPs 50%, 0.3 ms^−1^ for PVSPs 75% and 0.5 ms^−1^ for PVSPs 100% scenario over urban domain.

### Regional impact of photovoltaic solar panels on planetary boundary layer

**Figure 6:**
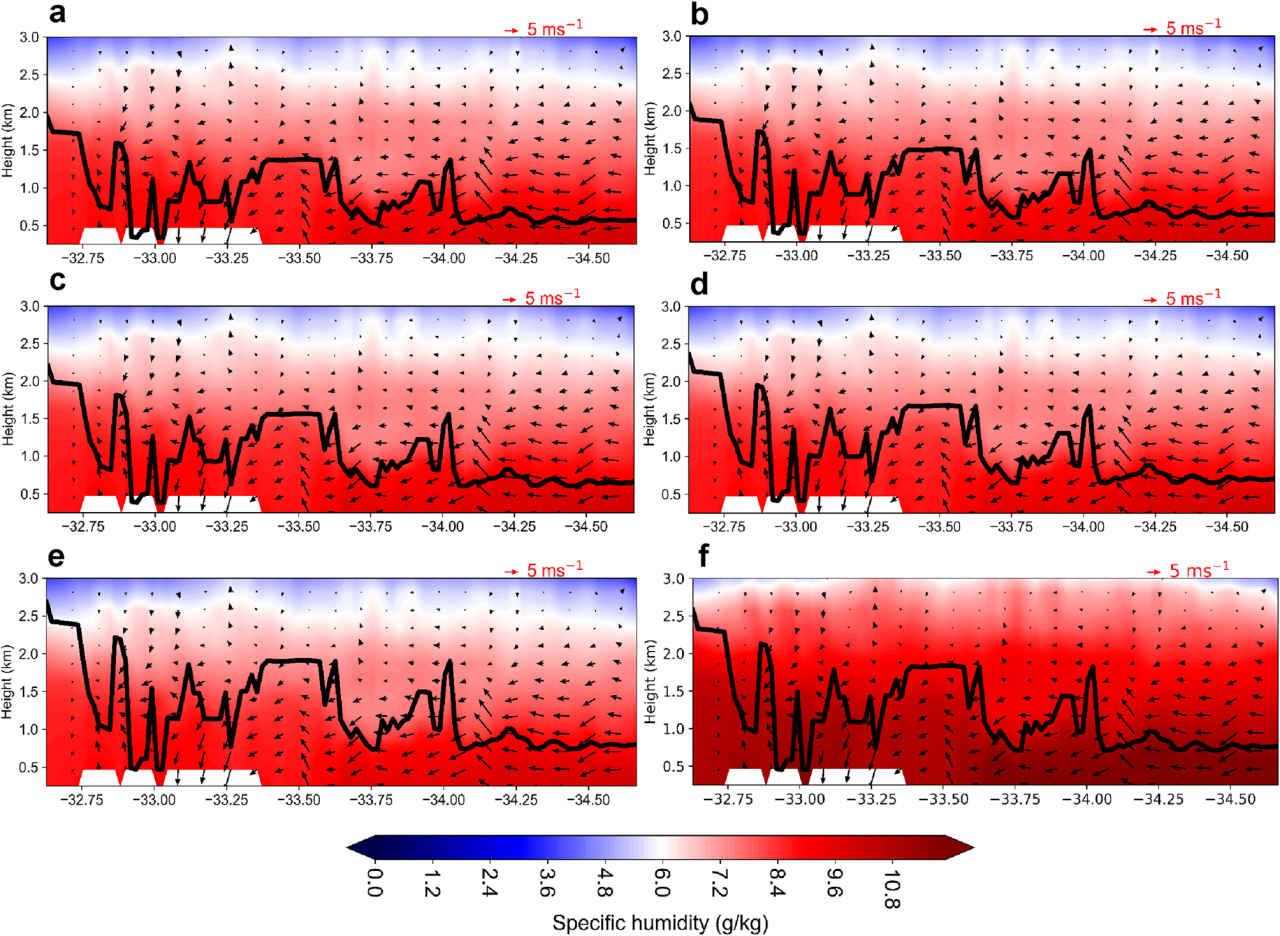
Cross-sectional profile of PVSPs impacts on sea breeze during peak hour (14:00 LT) over Sydney from east (sea breeze) to west (inland): (**a**) cool roofs (**b**) PVSPs 25%, (**c**) PVSPs 50%, (**d**) PVSPs 75%, and (**e**) PVSPs 100%, (**f**) vertical cross section showing the specific humidity difference between the PVSPs 100% and cool roofs simulations. The vertical gradient of specific humidity determines the static stability of the lower atmosphere. During high solar, the convective boundary layer developed the very fastest way and progressively increases with the implementation of PVSPs. This map was prepared by the first author with the help of WRF-Python (https://github.com/NCAR/wrf-python) and does not require any permission from anywhere.

The changes in planetary boundary layer (PBL) depth are seen in the Sydney urban region for each scenario as compared to the control scenario. It is evident that any coverage rate of large-scale PVSPs deployment increases the convective PBL height. The maximum increase for PVSPs 100% experiments ranges from 170 to 540 m, whereas the minimum increase for PVSPs 25% experiments ranges from 45 to 150 m. In addition, we examined the regional distribution of PBL height rise averaged across the full 59-day excessive heat period (for these WRF model experiments). The results demonstrate that the convective PBL depth rose over Sydney urban regions, indicating that the lower atmosphere has a greater ability to efficiently mix pollutants vertically, with possible consequences for low level pollutants concentration over ground level. Figure 6 depicts the complex and changeable geographical gradients seen over the urban domain of Sydney. The investigation demonstrated that these structures are reliant on horizontal grid size since comparable impacts were observed across the simulation periods, but an examination to verify their believability required further research and is beyond the scope of the current work.

The high-density urban building environment has an influence on lower atmospheric dynamics from the city to the regional scale. The diurnal fluctuation of the PBL was documented as a result of the effects of PVSPs technology at the city scale. When a higher fraction of PVSPs are implemented on rooftops at the city scale, then the level of the PBL height increases considerably. The regional distribution of the PBL height in the instance of PVSPs installation at 14:00LT is depicted in Fig. 6. The vertical wind speed distribution as a function of PBL height and available moisture in the lower atmosphere. For example, in the city’s core urban regions, the effects of PVSPs on PBL depth rise appear to extend beyond the scope of the deployment itself. The maximum increases of PBL height during 14:00 LT are 170.4 m, 256.2 m, 323.8 m, and 537.9 m for PVSPs 25%, PVSPs 50%, PVSPs 75% and PVSPs 100% respectively with average values are 74.6 m, 112.5 m, 139.6 m, and 233.2 m. At 18:00LT, the maximum increase of PBL height is 145.4 m for PVSPs 25%, 199.8 m for PVSPs 50%, 312.9 m for PVSPs 75%, and 451.8 m for PVSPs 100%. The highest rise is related with peak hour (14:00 LT) over the CBD, Inner West, Parramatta, and Southern Sydney. The highest decline, on the other hand, is reported for the Sydney domain’s outer west during dawn and sunset.

The primary reasons of PBL depth increase are the absorption of solar energy into PVSPs over the roof surface, which results in an increase in sensible heat and concomitant turbulence in the lower atmosphere. It is also highlighted that an increase in PVSPs is likely to accelerate static stability at the diurnal scale of the PBL depth. Modification of the PVSPs enhances the consequences of urban-induced warming and increases the intensity of convective mixing, hence increasing the PBL depth, with possible advantages for air pollution dilution and dispersion throughout the city domain. This would assume that the urban surface still has moisture to relinquish to the atmosphere, which may not be the case over the long term as the PVSPs aid in higher transfers of moisture and the surface is not storing as much moisture as a non-urbanized surface. To some extent, increased moisture transfer from the urban surface to the vertical layer developed by the adoption of PVSPs could also be a very short term beneficial to cloud formation processes, increasing the amount of precipitation in urban areas or their downwind environs.

### Regional impact of photovoltaic solar panels on sea breeze circulations

The city of Sydney being a coastal city will most definitely have complex transport characteristics. Sea breeze impacts will highly influence the regular convective boundary layer due to the moisture gradient in the urban atmosphere. The patterns of the Sydney’s ambient temperature distribution were found to be significantly dependent on synoptic meteorological conditions and the strength of the advection flows. High intensities of the urban heating phenomena were connected with the presence of a sea breeze in the eastern portions of the city, which lowers the temperature of the coastal zone, paired with westerly winds from the inland, which heat up the Sydney’s western zones. The high intensities of the oasis phenomena were accompanied with low wind speeds and a very feeble development of the sea breeze in the coastal area^23^. The intensification of sea breeze circulation is also reliant on the large-scale synoptic background, which is crucial in regulating the prevailing wind at the near-surface. In the vertical dimension, the study found that the height of the PBL in Sydney is directly related to the advection of the sea breeze. When PVSPs are adopted at the city scale, circulation can be quickened (Fig. 6). The PVSPs have the ability to raise the PBL height and cause localized circulation over Sydney’s dense urban areas. The results also show that the sea breeze arrived early in the afternoon (14:00 LT) as a result of the ‘regional low’ influence within the upper PBL and offshore synoptic wind flow above the PBL. To refill the buoyant cold air, the less-buoyant lighter warm air over the urban domain flows towards the suburbs. This is due to the fact that an increase in the PBL height and heat transfer could be a result in a stronger sea breeze circulation. The PVSPs can accelerate the sea breeze front by increasing the vertical lifting of urban thermals, transporting and dispersing low-level movements caused by warm advection, and transporting and dispersing low-level motions caused by warm advection. As a result, increasing the vertical wind speed by 0.3–0.5 ms^−1^ cause a greater buoyant over the urban domain where PVSPs are installed. This is due to a temperature gradient between the PVSPs and the surrounding air. This temperature difference can lead to the generation of convective flows, which are horizontal airflows caused by warm air rising and cooler air sinking. The surface roughness (z_0_) characteristics are being painstakingly calculated to maximize the results in order to be beneficial in drawing cold air from sea breezes to the surface due to mixing effects. Moreover, it is demonstrated that the influence of sea breeze is significantly magnified in high density residential areas. The surface roughness due to urbanization with high rise buildings could slow the advent of sea breeze, and possible counteracting the heating from the PVSPs to urban environments. Furthermore, the horizontal wind shear and frontal lifting caused by surface roughness characteristics may delay the arrival of the sea breeze front in the urban center. The efficacy of sea breeze advection is affected by the coastal location, shape and size of the city, which influences the urban heating impact caused by PVSPs. As a result, PVSPs for cities have significantly altered the thermal and dynamic profiles of the UBL and sea breeze circulation. This synoptic flow dominates in the parallel direction of the sea breeze, and the sea breeze fronts are less prone to secondary pollution deposition in the back of the front.

The study also demonstrates that implementing PVSPs on a city scale can change the pressure gradient between the city and the surrounding surface due to a considerable rise in ambient temperature of up to 1.4 °C and wind speed of up to 1.2 ms^−1^. Thus, fluctuations in PVSPs, sensible heating, and wind result in feedback within the city’s local climate during peak hours (14:00 LT). Higher PVSPs fractions enhance advective flow between the city and its environs, which increases local warming effects. It develops a 'regional low', which can increase both horizontal and vertical wind speed over the city. The average increase in wind speed in the north-west and south-west at 14:00 LT is 0.8 and 0.9 ms^−1^, respectively. As a result of the influence of this ‘regional low' over the domain, the rise in PVSPs may hasten the warm airflow from the nearby desert into western Sydney (Fig. 6). It can be expected that the collision of the warm dry desert with the cooler, humid air off the nearby ocean over the city result in significant exchanges of energy within the urban atmosphere over the city. In summary, the PVSPs impacts are as large as this modeling study shows, then there could be some significant knock-on effects over the urban atmosphere over Sydney.

## Discussions

**Figure 7:**
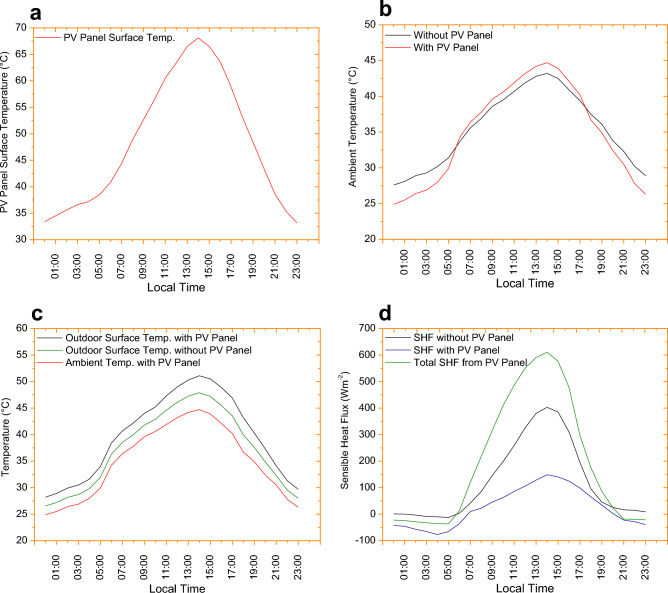
Substantiation of local warming in urban environments presented with two months average value for the heat wave period (January and February, 2017) when PVSP installed on the cool roofs: (**a**) average PVSP surface temperature; (**b**) average ambient temperature with and without PVSP effect; (**c**) average outdoor surface and ambient temperature with and without PVSP effect; (**d**) average sensible heat flux with and without PVSP effect and total average sensible heat flux from front and back of PVSP surface.

The PVSPs can increase local temperatures in dense urban environments due to a phenomenon called the ‘local urban warming effects’. When PVSPs are installed on rooftops in dense urban areas, they can absorb large amount of solar energy and convert it into less amount of electricity. Therefore, PVSPs hold heat and release it slowly into the local urban environment via convection. As a result, the temperature of the PVSPs can become higher than the surrounding ambient temperature, and this can lead to an increase in local temperatures. In addition, the heat absorbed by the PVSPs can be transferred to the building underneath, further increasing the building’s temperature. PVSPs can reach their panel surface temperatures of up to 70 °C due to several factors related to their operation and design. These factors include: solar radiation, wind speed, convective heat transfer, efficiency, design, orientation and shading. As a result, heat is transmitted from the top and bottom surfaces of the PVSPs away. Due to the local wind speeds, convection coefficients may be slightly higher on the upper PVSPs surface. Figure 7 shows substantiation of local warming in urban environments presented with two months average value for the heat wave period (January and February 2017) when PVSPs 100% installed on the cool roofs. In the study, we demonstrate that the PVSPs surface temperature may exceed 68.1 °C during the peak hours of the day if we installed 100% PVSP on the cool roofs of urban building in Sydney (Fig. 7a). The primary reason of this higher temperature is convective heat transfer from both PVSPs surfaces to the surrounding air over the urban domain. Figure 7b illustrates how cool roofs, as opposed to traditional black roofs, might increase the effects of PVSPs on urban areas. The findings show unequivocally that, in comparison to PVSPs roofs, the urban ambient temperature increased by up to 1.5 °C during the hottest peak hour. This primarily happened because of realistic depictions of the PVSPs energy balance. Also, it was found that large scale PVSPs during a typical hot day in Sydney caused the air temperature to increase by 1.5 °C during the day and decrease by 2.7 °C at night. Thus study infers that the roof surface was less warmed by longwave radiation trapped between the shadowed roof and PVSPs caused by cool roof effects, increasing the ambient air temperature is only due PVSPs convective heat transfer in outdoor urban environments. Hence, the PVSPs should continue to be cooler than the surrounding air for the bulk of the night due to poor thermal storage and strong thermal emissivity. The increasing ambient temperature during the day is impacted by the PVSPs’ efficiency (ambient temperature penalties) and cool roofs. Hence, adopting cheap, readily available, and inefficient PVSPs could reduce overall solar reflectance, increasing the risks of urban heating by PVSPs, particularly during the daytime.

At peak hours (14:00), the surface temperature increases by 2.5 °C and during night decrease by 3.4 °C (Fig. 7c). This is because of offset between ambient air temperature and surface temperature in WRF model depends on various factors. WRF model strives to simulate the temporal dynamics of both surface and air temperatures over urban domain, discrepancies between the two can arise due to limitations in the representation of heat transfer processes, surface characteristics, atmospheric mixing and advection, and solar forcing. These discrepancies may be related to uncertainties in model physics, parameterizations, or boundary conditions. Additionally, regional or local factors such as topography, land use, or vegetation cover can introduce further complexities that affect the relationship between surface and air temperatures. The surface temperature could increase as a result of the PVSPs’ local warming effects due to a process called heat transfer. Remembering that the surface temperature is affected by factors other than the ambient temperature is important. The solar irradiation, the wind velocity, and the PVSPs design are a few more factors that influence the surface temperature. Peak sensible flux was significantly reduced when a cool roof took the place of a black roof. However, the peak flux is more affected by the installation of PVSPs to a cool roof, although the sensible heat flux is increased by 170.9% (Fig. 7d). Sensible heat flux is decreased to 148.6 Wm^−2^, -when cool roof installation is implemented on a standard conventional roof. But after adding PVSPs to a cool roof, the sensible heat flux increased to 402.6 Wm^−2^, when used in Sydney’s urban settings, PVSPs performs like typical black roofs and subsequently increase local warming. According to the findings, 100% PVSPs roof would enhance total sensible flux if a cool roof were to be replaced. This is typically caused by the total sensible heat flux from the PVSPs surface’s front and rear through heat convection added into the urban environments up to 610.6 Wm^−2^ in Sydney.

**Figure 8:**
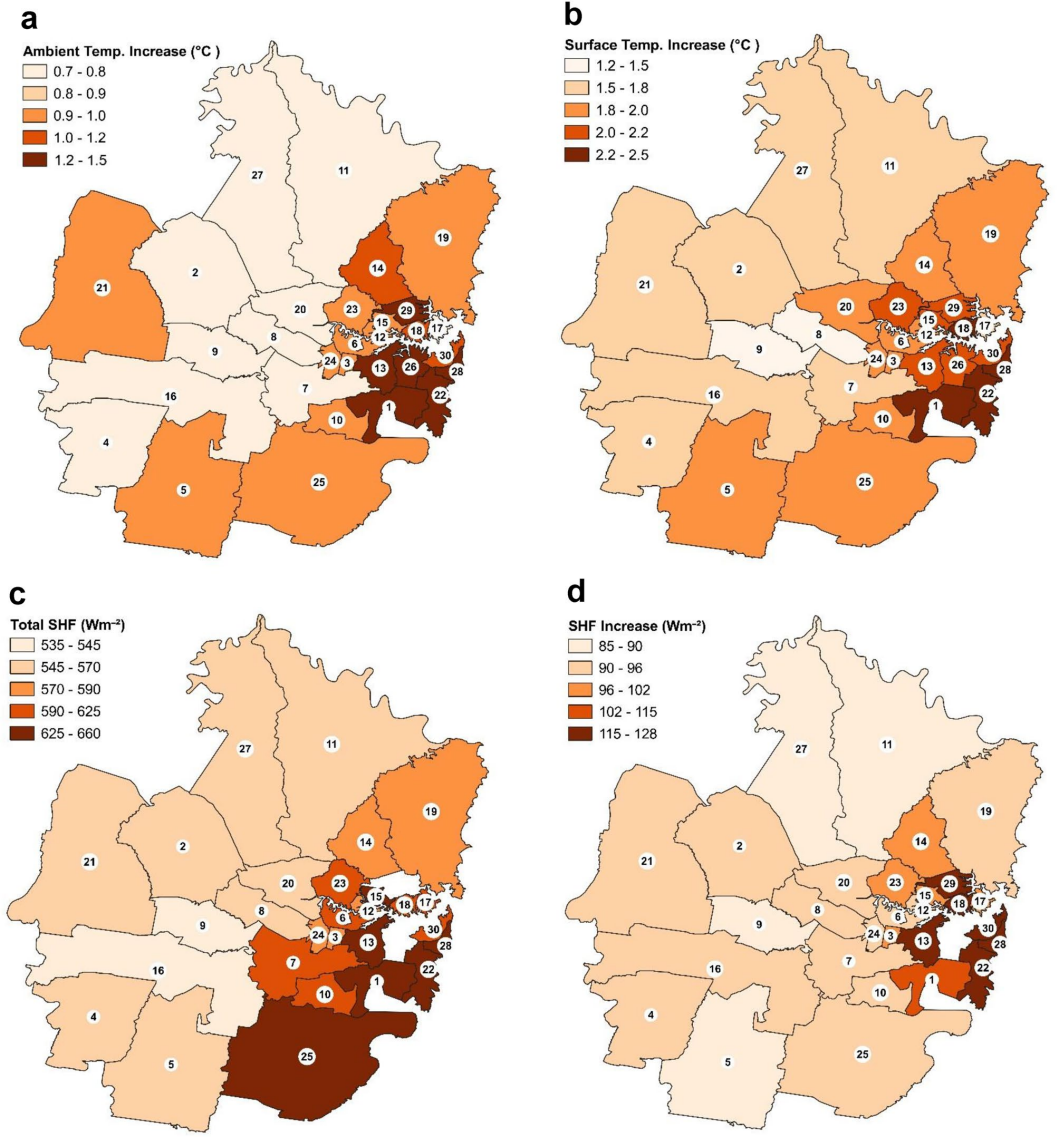
(**a-d**) Spatiality of local warming in urban districts presented with average value for a typical hottest day when PVSP installed on the cool roofs: (**a**) daytime average increase in ambient temperature (with PVSP minus without PVSP); (**b**) daytime average increase in surface temperature (with PVSP minus without PVSP); (**c**) Release of total sensible heat flux from front and back of PVSP surface during peak hour; (**d**) Increase in 24-h average sensible heat flux (with PVSP—without PVSP). The urban district is identified by code: (1) Bayside, (2) Blacktown, (3) Burwood, (4) Camden, (5) Campbelltown, (6) Canada Bay, (7) Canterbury-Bankstown, (8) Cumberland, (9) Fairfield, (10) Georges River, (11) Hornsby, (12) Hunters Hill, (13) Inner West, (14) Ku-ring-gai, (15) Lane Cove, (16) Liverpool, (17) Mosman, (18) North Sydney, (19) Northern Beaches, (20) Parramatta, (21) Penrith, (22) Randwick, (23) Ryde, (24) Strathfield, (25) Sutherland, (26) Sydney, (27) The Hills, (28) Waverley, (29) Willoughby, and (30) Woollahra. This map was prepared by the first author with the help of ArcGIS 10.4 (http://www.esri.com/software/arcgis) and does not require any permission from anywhere.

Spatiality of local warming in 30 urban districts is shown with average values for a typical hottest day when PVSPs were installed on the cool roofs (Fig. 8a–d): (a) increase in average daytime ambient temperature (with PVSPs minus without PVSPs); (b) increase in average daytime surface temperature (with PVSPs minus without PVSPs); (c) release of total sensible heat flux from the front and back of PVSP surface during peak hour; (d) increase in average 24-h sensible heat flux (with PVSPs—without PVSPs). In the urban districts of Ryde, Willoughby, Lane Cove, Mosman, Canada Bay, North Sydney, Woollahra, Inner West, Sydney, Waverley, Randwick, Srathfield, and Bayside, the average increase in ambient temperature ranges from 0.9 to 1.5 °C is shown in Fig. 8a. In the urban areas of North Beaches, Ku-ring-gai, Parramatta, Ryde, Willoughby, Lane Cove, Mosman, Canada Bay, North Sydney, Woollahra, Inner West, Sydney, Waverley, Randwick, Georges River, Sutherland, Campbelltown, and Bayside, the average increase in surface temperature ranges from 1.8 to 2.5 °C is shown in Fig. 8b. In the urban districts of North Beaches, Ku-ring-gai, Ryde, Willoughby, Lane Cove, Mosman, Canada Bay, North Sydney, Woollahra, Inner West, Sydney, Waverley, Randwick, Georges River, Sutherland, Canterbury-Bankstown, and Bayside, the release of total sensible heat flux from the front and back of PVSPs surface during peak hour ranges from 571 to 660 Wm^−2^ is shown in Fig. 8c. According to Fig. 8d, the increase in the 24-h average sensible heat flux (with PVSPs versus without PVSPs) for the urban areas of Ku-ring-gai, Ryde, Willoughby, Lane Cove, Mosman, Canada Bay, North Sydney, Woollahra, Inner West, Sydney, Waverley, Randwick, Strathfield, and Bayside ranges from 97 to 128 Wm^−2^. High density urban areas tend to show higher warming due to a combination of additional factors including the urban heating due to local climate change, lack of green or blue spaces, high energy use, urban metabolism and human activity can contribute to the higher temperatures seen in high density urban areas of the Sydney.

**Figure 9:**
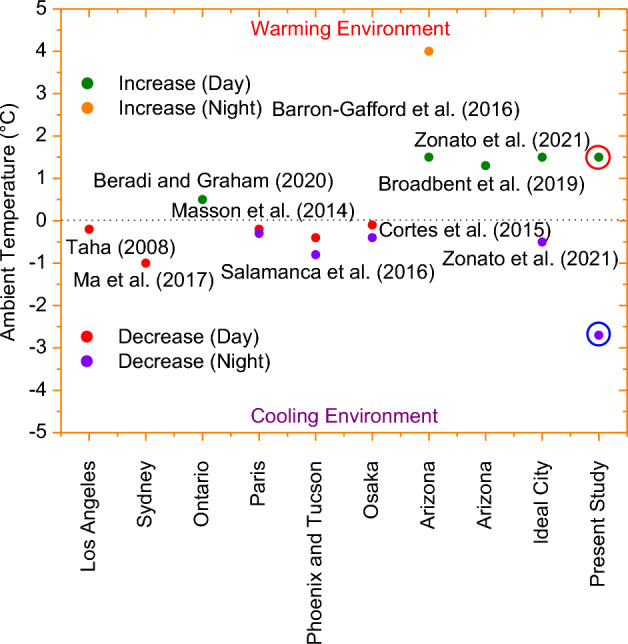
PVSPs have an effect on the energy balance of cities, which in turn may have an impact on air temperatures. Notwithstanding the fact that this corpus of literature contains inconsistent outcomes, review and synthesis point to two important conclusions. PVSPs have the ability to considerably warm the city during the diurnal scale, and PVSPs can also likely cool the urban environment on a diurnal scale. Nonetheless, the results of the current study indicated that while PVSPs can significantly cool the city at night because of the impacts of cool roofs, they can also significantly warm the city during the day due to heat convection.

Even though there is some disagreement in the previous studies, the majority of experimental data and more recent, thorough modeling that takes into account longwave exchange between PVSPs surface and urban surfaces, convective heat transfer from both sides of PVSPs, and other advancements suggest that current PVSP implementations in urban settings could probably have a warming effect on ambient temperatures during the day and a cooling effect at night^1^. In this section, we summarized some reported studies that cool or heat cities in various urban or suburban environments (Fig. 9). Many studies have reported that PVSPs can help to cool the urban environment in Log Angeles, Sydney, Paris, Phoenix and Tucson, and Osaka^3,5,9,10,24^. This is primarily due to simplistic representations of the energy balance of PVSPs in urban environments. However, very few studies have found that PVSPs can increase local temperatures when the realistic PVSPs’ energy balance is taken into account in Ontario^6^, Arizona^2^, Arizona^13^ and, Ideal City^19^. Because PVSPs installation and thermal properties play a significant role in how much of the impact they have on urban ambient temperatures. According to Zonato et al. (2021)^19^, PVSPs cause a temperature increase of 1.5 °C during the day and a slight decrease of 0.5 °C at night. The PVSP has a lower thermal inertia than the roof surface because of its thinness (6.55 mm). Because of this, the ambient temperature for PVSPs increases throughout the day and is higher in cramped, tiny spaces. Furthermore, the ambient temperature at night is lower in the case of cool roofs with PVSPs due to the cool roofs’ daytime reflectivity and the shading effect of the PVSPs, which both prevent heat from being held on the roof surface during the day and then released negligibly at night. On top of that, roof surface temperature under PVSPs are warmer at night than cool roofs without PVSPs because cooling due to longwave emission are blocked by the PVSPs. PVSPs reduce outdoor air temperature during night because PVSP has very lower thermal inertia and their rapid cooling cools the surrounding air by convection. The ambient temperature increase during the day is remarkably similar to our Sydney findings i.e. 1.5 °C. However, because of local weather conditions and cooling effects of cool roof, nighttime drops are very considerable. According to a microscale atmospheric modeling study in Ontario, Canada, the widespread deployment of PVSPs’ rather than cool (white) roofs will result in outdoor warming penalty. In particular, Berardi and Graham (2020)^6^ found that rooftop PVSPs might result in a 0.5 °C urban warming. However, the study also included an effective albedo technique representation of PVSPs and assumed that they were flush with the roofs. Another observational analysis found that, in southern Arizona, near PVSPs’ arrays, the daily average air temperature was 1.3 °C warmer than at a comparable reference site without PVSPs^13^. A large-scale PVSPs module array was shown to increase ambient temperature by 1.5 °C during the day and 3 to 4 °C at night in a distinct experimental evaluation carried out in southern Arizona^2^. Our Sydney study’ findings on the urban daytime increase are astonishingly similar to these two studies. According to Barron-Gafford et al. (2016)^2^, longwave radiation trapped between the PVSPs’ and the shaded ground warmed the ground surface, increasing the temperature of the surrounding ambient environments. However, as demonstrated by Pham et al. (2019)^8^, the PVSPs’ should continue to be cooler than the surrounding ambient air for the majority of the night due to their low thermal storage and high thermal emissivity. While a warming of the ambient air during the daytime is typical, a warming during the night is more unexpected. The study’s quantify of urban warming is therefore probably too negligible. Scherba et al. (2011)^25^ conducted simulation studies to examine the effects of PVSPs installation over three various roof types: a white roof with a solar reflectance of 0.7, a green roof with vegetation, and a black roof with a 0.06 solar reflectance and a leaf area index of 1.0. They found that roof-mounted PVSPs’ on a very dark roof led to less overall warming of the urban airshed than an unshaded, very dark roof alone (with solar reflectance of 0.06). However, they did find that adding PVSPs’ to a roof that was lighter in color (with a solar reflectance of 0.7) caused a significant warming of the urban airshed. These findings are very similar to present study because we used PVSPs on highly reflective cool materials (0.8).

## Conclusions

The PVSPs in urban environments have a lot of advantages, but they also have certain drawbacks, both in terms of how the urban environment affects their performance and how PVSPs potentially overheat the urban environment. These complexities can be difficult to communicate to urban planners or decision-makers, who are typically looking for succinct explanations of PVSPs efficiency. When communicating the negative effects of any sustainability solution, the scientific community must exercise caution because they are sometimes taken out of context and can significantly slow the penetration of technologies that, despite their limitations, continue to benefit society as a whole; urban PVSP is no exception. The force and characteristics of the PVSPs are heavily influenced by synoptic meteorological conditions, notably the development of the sea breeze and westerly winds from the desert area is Sydney. The possibility of a secondary heating mechanism, such as advection of warm air from neighboring places, might exacerbate the severity of the situation when PVSPs implemented on rooftop. At city-scale, an increase in PVSPs can raise peak summer ambient temperatures by up to 1.4 °C and surface temperatures by up to 2.3 °C. Temperature disparities between the eastern and western areas of the city were observed during the study period. The patterns of the city’s ambient temperature distribution were determined to be highly reliant on synoptic meteorological conditions and advection flow strength. In addition, local warming effects of PVSPs were observed at urban district-scale. The large-scale deployment of PVSPs at local district-scale during a typical hot day in Sydney caused the air temperature to rise by 1.5 °C during the daytime and decrease by 2.7 °C at nighttime. The maximum increase of sensible heat and latent heat flux was 245.5 Wm^−2^ and 11.5 Wm^−2^, respectively. Wind velocity can be increased by up to 1.2 ms^−1^. As a result, larger urban PVSPs values promote advective flow between the city and its environs. Modification of the PVSPs in Sydney leads in a significant rise in PBL heights over the city of up to 537.9 m and may reduce pollutant concentrations at ground level. The advent of a sea breeze in the city’s eastern portions, which reduces the temperature of the coastal zone, combined with inland westerly winds, which heat the city’s western zones, decreased the severity of the UHI phenomenon due to warming effects of PVSPs.

In addition, future research could be focused on enhancing the reflectance of solar energy wavelengths that are not converted to energy. PVSPs may play an important role in our future energy mix as we work to reduce green house-induced global warming. However, we believe that panels that reject non-transformed heat more efficiently might be developed. For the fraction of the PVSPs spectrum that cannot be turned into electricity, further research may provide PVSPs systems that are more reflective of these wavelengths or more strongly emissive of their own energy. Consider some recent advances in material science known as the photo thermal conversion plates on the surface of PVSPs are made of highly absorbent materials, so as to improve the efficiency of photo thermal conversion. By merging some of these material science findings with traditional PVSPs breakthroughs, a new generation of ‘cool photovoltaic’ will be developed. So, PVSPs that are as efficient as our present generation of PVSPs but far more efficient thermally, allowing them to operate at significantly lower temperatures. Furthermore, because of the temperature coefficient, they would very definitely benefit from an extra efficiency increase by operating at cooler temperatures and expelling less heat into the urban environment. Additionally, to evaluate the impact of PVSPs, conducting a field study at a building scale and incorporating the findings into a mesoscale model to understand the city-wide impacts would be highly desired in future study.

## Methods

This paper attempts to bring attention to this issue and initiate scientific discussions within the PVSP community. So certain aspects are beyond the expertise as well as the scope of this general work. We will surely incorporate these as more progress is made. However, there are many limitations even in observations as well as model.WRF model works at large spatial scale and hence even in an urban model, most interactions are to a large extent averaged or parameterized. It is also difficult to incorporate all the processes including PVSP albedo, heat storage and other aspects as such measurements even for parameterization are not available for our conditions.

To resolve these issues in WRF model, Zonato et al., (2021)^19^ have updated the urban storage heat flux in the WRF model to explicitly incorporate the urban energy balance. They also tested PVSP in the WRF-BEP + BEM model with this newly parameterized urban storage heat flux. Their results reveal that the model explicitly captures the effects of PVSP on the urban environment. Therefore, we used their parameterized schemes for the present investigation at the city scale. To account for the effects of PVSPs inside BEP + BEM, the parameterization provided in this study assumes that PVSPs arrays are parallel, detachable from roofs, and include a single layer^19^. Unlike Masson et al. (2014)^10^ and Salamanca et al. (2016)^9^, they parameterized the PVSPs temperature based on its dependency on shortwave solar radiation, while Zonato et al. (2021)^19^ explicitly developed the PVSPs temperature that relies on all of the related components of wavelength. Once temperature is calculated, the value of the incoming heat flux is updated and sent to the multilayer urban canopy scheme. Zonato’s schemes^19^ were used to assess the impacts of PVSPs in the study. This study is being done in Sydney, Australia, to investigate the environmental effects of PVSPs. The breadth and characteristics of a strong urban heatwave in Sydney were investigated using mesoscale WRF model.

### *Assumptions of PVSPs within WRF/BEP* + *BEM*

**Figure 10:**
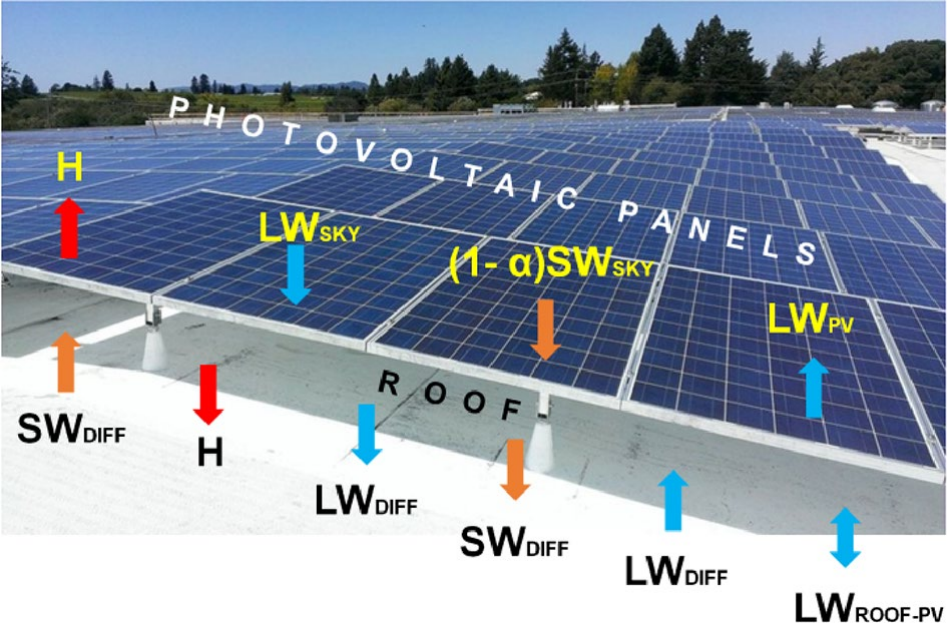
The PVSP design in WRF/BEP + BEM^19^, with a schematic representation of the energy exchanges with the underlying cool roofs and the ambient environment. When calculating the radiative heat exchange with the other elements, the PVSPs are assumed to be horizontal in geometry: exchanges between the roof, the PVSPs, and the sky above are assumed to be solely vertical. We take the inclination of the PVSPs into consideration when calculating the irradiance for electricity generation. The energy balance equation of the PVSP is found in methods section. The parameterization employed in this study assumes that PVSPs arrays are parallel, unattached from roofs, and made of a single layer in order to account for the impacts of rooftop PVSPs inside WRF/BEP + BEM schematically shown in Fig. 10.

The parameterization employed in this study assumes that PVSPs arrays are parallel, unattached from roofs, and made of a single layer in order to account for the impacts of rooftop PVSPs inside WRF/BEP-BEM schematically shown in Fig. 10. The time derivative of PVSPs temperature ($T_{pv}$) reads with all terms in ${\text{Wm}}^{ - 2}$^19^:

1 $$C_{{{\text{module}}}} = \frac{{\partial T_{pv} }}{\partial t} = \left( {1 - \alpha_{pv} } \right)sw_{sky}^{ \downarrow } + \varepsilon_{pv}^{U} LW_{sky}^{ \downarrow } - LW_{pv}^{ \uparrow } + LW_{roof - pv}^{ \updownarrow }$$

2 $$- E_{pv} - H^{ \uparrow } - H^{ \downarrow }$$

3 $$+ \left( {1 - VF} \right)\left[ {\left( {1 - \alpha_{pv} } \right)SW_{diff} + LW_{sky}^{ \downarrow } } \right]$$

### Details of terminologies

- $C_{{{\text{module}}}}$ : The corresponding heat capacity per unit area is 5*.*72 MJK^−1^ m^−2^, assuming that the PVSPs is comprised of three layers, as described by Jones and Underwood (2002)^26^: a monocrystalline silicon PVSPs cell, a polyester trilaminate, and a glass face, with a total depth of 6.55 mm. Jones and Underwood (2002)^26^ provide the heat capacity, depths, and density values for each stratum^19^.
- $\left( {1 - \alpha_{pv} } \right)sw_{sky}^{ \downarrow }$: Assuming an albedo is $\alpha_{pv}$ = 0.11, the net shortwave radiation gained by the PVSP’s upward surface^19^.
- $\varepsilon_{pv}^{U} LW_{sky}^{ \downarrow }$ : Incoming longwave radiation near the PVSP’s top surface, where $\varepsilon_{pv}^{U}$ = 0.79 is the emissivity of the glass face^19^.
- $LW_{pv}^{ \uparrow } = \varepsilon_{pv}^{U} T_{pv}^{4}$ : The PVSPs emits upward longwave radiation^19^.
- $LW_{roof - pv}^{ \updownarrow } = VF\frac{1}{{\frac{{1 - \varepsilon_{pv}^{D} }}{{\varepsilon_{pv}^{D} }} + \frac{{1 - \varepsilon_{roof} }}{{\varepsilon_{roof} }}}}\sigma \left( {T_{pv}^{4} - T_{roof}^{4} } \right)$: Longwave radiation exchanged between the PVSP’s downward monocrystalline silicon face ($\varepsilon_{pv}^{D}$ = 0.95) and the upward face of the roof. The radiation fluxes from the PVSPs and the roof are examined jointly to account for the numerous reflections between the two surfaces. The view factor between the PVSP’s downward face and the ceiling is denoted by VF. Assuming a 10 m × 10 m PVSP (covering completely the roof, with a clearance of 0.3 m from the underlying surface, VF = 0.06)^19^.
- $E_{pv} = \eta_{pv} sw_{sky}^{ \downarrow } \min \left[ {1,1 - 0.005\left( {T_{pv} - 298.15} \right)} \right]$ : The PVSPs generates energy in presence of sunlight. It considers that the efficiency of PVPs diminishes at temperatures over 25 °C;$\eta_{pv}$ is the PVP’s conversion efficiency, or the proportion of shortwave radiation converted into electricity. The efficiency of quantum dot cells ranges from 7 to 44% when employed in research applications^27^. Because the most frequent rooftop arrays are monocrystalline and polycrystalline silicon PVSPs, we use an efficiency $\eta_{pv}$ = 0.19^19^.
- $H^{ \uparrow } + H^{ \downarrow } = \left( {h^{ \uparrow } + h^{ \downarrow } } \right)\left( {T_{pv} - T_{air} } \right)$: The sensible heat fluxes on the PVP’s upper and downward faces. The formulation for $h = \sqrt {h_{c}^{2} + a\left| V \right|^{b} }$ depends on empirical fits and is adopted from the EnergyPlus model^28^, which has been validated against measurements^25^. The term $h_{c}$ is determined by the surface material (glass in this example), whether the surface is facing upward or downward, and the sign of the temperature differential between the surface and the air. The absolute wind speed is taken at the first level of the WRF above the roofs, and it is assumed to be the same for the upward and downward faces^19^.
- $\left( {1 - VF} \right)\left[ {\left( {1 - \alpha_{pv} } \right)SW_{diff} + LW_{sky}^{ \downarrow } } \right]$ : The downward PVSPs surface receives diffuse shortwave and longwave isotropic radiations.$LW_{sky}^{ \downarrow }$ the sky is the incoming longwave radiation, while $SW_{diff}$ is the diffuse shortwave radiation. The roof below the PVSPs receives the same amount of diffuse shortwave and longwave radiation^19^.

Unlike Masson et al. (2014)^10^ and Salamanca et al. (2016)^9^, the parameterized $T_{pv}$ through its dependence on shortwave solar radiation, But, Zonato et al. (2021)^19^ numerically unravel Eq. (1), in a way similar to Jones and Underwood (2002)^26^, to get a PVSPs temperature that depends on all the involved contributions. Once $T_{pv}$ is calculated, the value of the outgoing heat flux is updated and passed to the multilayer urban canopy scheme^19^.

### Model configuration

**Figure 11:**
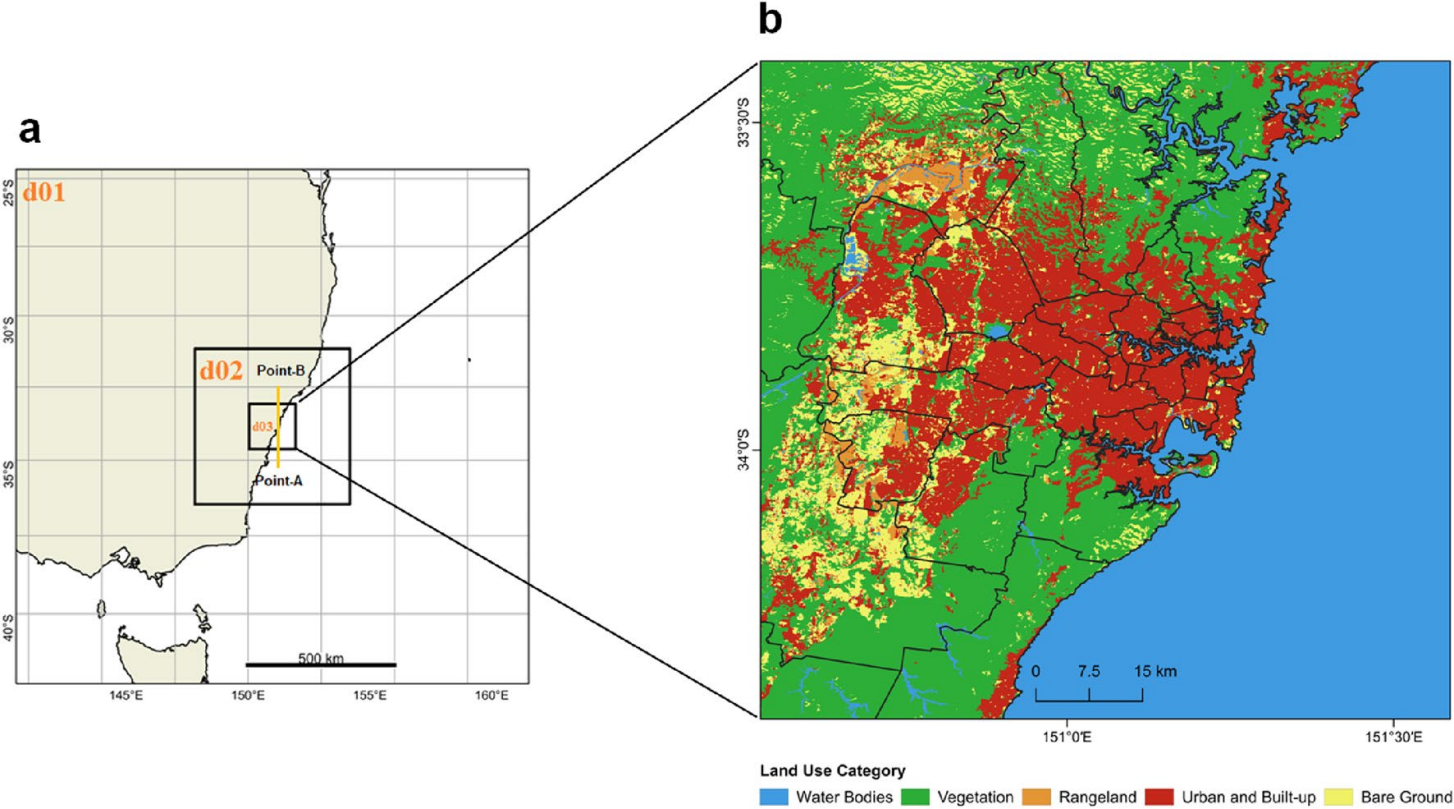
WRF domain shows (**a**) dynamical downscaling with domain 1 (d01) as the outermost parent domain with 4.5 km grid spacing, domain 2 (d02) with 1.5 km grid spacing, and, an innermost domain 3 (d03) with 0.5 km grid spacing; innermost d03 with 0.5 km grid spacing which encompasses the Greater Sydney). Point-A (east) and Point-B (west) are the points used for drawing horizontal-vertical cross-sections to analyze meteorological conditions for Fig. 6 and (**b**) The map of updated land use/land cover (LULC) derived from ESA Sentinel-2 imagery at 10 m resolution for the innermost domain. This map was prepared by the first author with the help of WRF-Python for WRF domain (https://github.com/NCAR/wrf-python) and ArcGIS 10.4 for LULC map (http://www.esri.com/software/arcgis) and does not require any permission from anywhere.

We employ a comprehensive mesoscale climatic model for the entire city of Sydney, which is an advanced frequently used weather research and forecasting (WRF v4.3.1) model^29^ coupled to the multilayer building parameterization and energy (BEP + BEM) system^30^. The ability of the BEP + BEM system to replicate the observed diurnal cycle of near-surface variables (ambient air temperature, wind speed, and wind direction) and city wide energy consumption at the city scale^31^. The Noah land-surface model^32,33^ was used for the proportion of grid cells with natural cover, while the multilayer building parameterization, and energy model was used for the fraction with built cover. The model is created to approximate the distribution of the city’s key climatic conditions under all meteorological, synoptic, and land use situations. To assess the regional impacts of large-scale PVSPs deployment on near-surface standard meteorological fields and urban climate, we conduct one control and four PVSPs scenario with high resolution WRF model experiments, each covering the 2-month clear-sky extreme heatwave period from 1 January 12:00 h, 2017 to 28 February 00:00 h, 2017. All WRF model simulations were started twelve hours before (i.e., 31 December 00:00 h, 2016), and this time interval was designated the model spin-up phase. The horizontal domain was made up of three two-way nested domains with grid points at 200 × 200 (d01), 202 × 202 (d02), and 202 × 202 (d03) with spatial resolutions of 4.5 km, 1.5 km, and 0.5 km, respectively (Table 1, and Fig. 11). The inner domain (d03) encompasses practically the whole city of Sydney, including the urban areas. For initial and boundary conditions, all WRF model experiments used ERA-Interim data. The map of updated land use/land cover (LULC) derived from ESA Sentinel-2 imagery at 10 m resolution for the innermost domain. The ESA Sentinel-2 imagery LULC categorization was used to generate non-urban LULC classifications along with updated urban and built-up land overlaid on Sydney city. Urban environmental factors can have a significant impact on the performance of PVSPs in cities. Here are some ways in which urban environmental factors influence the performance of PVSPs such as land use, building height, roof width, heat capacity of buildings, thermal conductivity of buildings, albedo and emissivity of buildings, street parameters, anthropogenic heat, etc. The parameters in WRF urban parameter table may vary greatly from city to city. The default values are most likely not appropriate for Sydney city. Thus these values based on the Sydney city have been adapted in WRF urban parameter table to replicate the actual effects of urban environmental factors on PVSPs performance.

**Table 1:** WRF/BEP + BEM model configuration.

Configuration	Domain 01 (d01)	Domain 02 (d02)	Domain 03 (d03)
Version	ARW-WRF v4.3.1		
Initial and boundary conditions	ERA-Interim reanalysis		
Run time	31 December 00:00 h, 2016 to 1 March 00:00 h, 2017		
The period for analysis	1 January 00:00 h, 2017 to 28 February 00:00 h, 2017		
Grid distance (m)	4500	1500	500
Grid number	200 × 200	202 × 202	202 × 202
Number of vertical layers	40 layers		
Microphysics	WRF single-moment 6-class scheme		
Surface layer model	Noah-LSM + BEP + BEM)^30^		
Turbulence	TKE scheme^34^		
Short-wave radiation	Dudhia scheme^35^		
Long-wave radiation	RRTM scheme^36^		
Planetary boundary layer	Bougeault-Lacarrère (BouLac) PBL scheme^37^		
Cumulus parameterization	Kain-Fritsch (KF) scheme^38^

### Numerical design and experiments

The control WRF model experiment (hereinafter referred to as control scenario) was carried out by setting the albedo of the roofs to 0.80 and the proportion of the roofs covered by PVSPs to zero. The other four WRF model sensitivity studies relate to varied coverage rates of either roofing methods, when analyzed alone or together. The built mesoscale model is used to simulate the hourly distribution of important meteorological parameters in Sydney under current heatwave conditions, as well as four PVSPs scenarios. The PVSPs were installed in a uniform proportion across all urban grid cells. The PVSPs were evaluated at the urban scale on 25%, 50%, 75%, and 100% roof surfaces (Table 2). We did extensive investigation into the PVSPs scenario’s performance and heating potential. This study examines four PVSPs measures at the city level. For example, the PVSPs 0.25 WRF model experiment shows a hypothetical situation in which 25% roof of city is covered with PVSP (PVSPs 25%). Similarly, the WRF model simulation with fraction of PVSPs 0.50, PVSPs 0.75, and PVSPs 1.00 presents a hypothetical circumstance in which PVSPs cover 50% (PVSPs 50%), 75% (PVSPs 75%), and 100% (PVSPs 100%), respectively on the building  rooftop of the city. All WRF model tests using PVSPs arrays were carried out with the PVSPs albedo, and conversion efficiency set to 0.11, and 0.19, respectively, which are typical values for modern PVSPs technology^4,10,19,27^. PVSPs characteristics, such as albedo, inclination, and tilt elevation, are found in detailes^19^ in section of ‘assumptions of PVSPs within WRF/BEP + BEM’.

**Table 2:** WRF/BEP + BEM model designs and experiments.

Experiments	Purpose	Photovoltaic panel fraction	Thermal characteristics of roof and photovoltaic panel					
			Albedo		Emissivity			Conversion efficiency $\eta_{pv}$
			Roof $\alpha_{roof}$	Panel $\alpha_{pv}$	Roof $\varepsilon_{roof}^{U}$	Upward panel $\varepsilon_{pv}^{U}$	Downward panel $\varepsilon_{pv}^{D}$	
SR	Validation	0.00	0.20	0.00	0.85	0.00	0.00	0.00
CR	Control	0.00	0.80	0.00	0.85	0.00	0.00	0.00
PVSP 25%	Scenario	0.25	0.85	0.11	0.85	0.79	0.95	0.19
PVSP 50%	Scenario	0.50	0.85	0.11	0.85	0.79	0.95	0.19
PVSP 75%	Scenario	0.75	0.85	0.11	0.85	0.79	0.95	0.19
PVSP 100%	Scenario	1.00	0.85	0.11	0.85	0.79	0.95	0.19

*SR* standard roof, *CR* cool roof, *PVSP* photovoltaic solar panel.

### Model evaluation and validation

**Figure 12:**
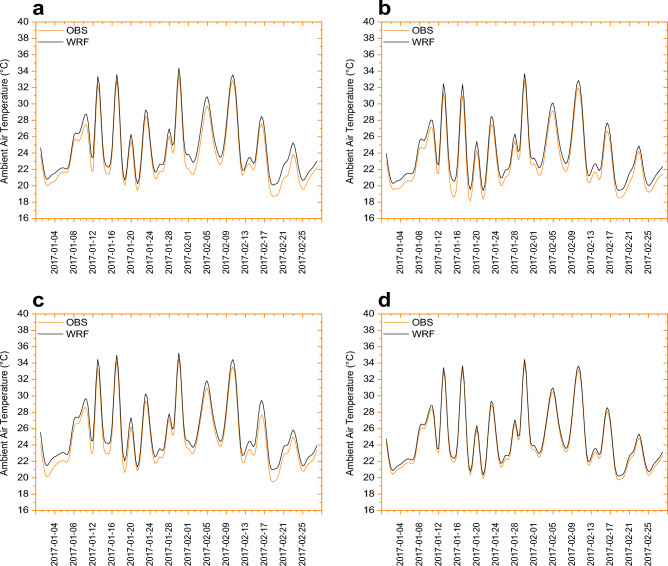
Validation of the WRF model and the corresponding observed ambient air temperature for the 24-h average duration for four local meteorological stations: (**a**) Penrith, (**b**) Observatory park, (**c**) Sydney airport, and (**d**) Olympic park.

In this study, we validated the model using conventional roofs (e.g., roof albedo = 0.2) rather than cool roofs. The standard roof (0.20) for PVSPs cases in Sydney in the current validation was based on the same simulation period as our previous two studies^20,39^ against the most representative Sydney’s current scenario (buildings with standard roofing system). WRF simulation results with regular or standard roofs (e.g., roof albedo = 0.2) were validated against neighboring meteorological stations before going on to PVSPs scenarios. Garshasbi et al. (2023a; 2023b)^20,39^ provide details on model validation for the same period as the control scenario. To assess the efficacy of the WRF/BEP + BEM system, we compared hourly simulated 2-m ambient air temperature to local observations for the control scenario simulation over urban grid cells in the innermost area in Fig. 12. Table 3 provides a statistical comparison of the mean bias error (MBE), mean absolute error (MAE), root mean square error (RMSE), correlation coefficient (R), and index of agreement (IOA) for hourly 2-m ambient air temperature during a 24-h period. The model is evaluated based on the correlation between the WRF model and data for 2-m ambient air temperature across the diurnal cycle. The combined WRF/BEP + BEM model accurately reflects the temperature recorded at four stations (mean R = 0.982; mean bias = 0.569) for Penrith (rural), Observatory park (urban), Sydney airport (urban), and Olympic park (sub-urban). The control scenario simulation reproduced urban meteorological conditions that were statistically consistent with local observations (*p* < 0.05). As a function of the prevailing local meteorological conditions, the simulated average UHI intensity ranged from 3.2 to 6.3 °C in high-density urban residential areas versus variable rural (i.e., surrounding) landscapes. The MBE and RMSE of air temperature were 0.4–0.9 °C and 0.5–0.9 °C, respectively. When all observation stations were included, the IOA ranged from 0.95 to 0.97, with an average value of 0.96.

**Table 3:** Comparison of the simulation results with observation data at an average of 24-h scale for 59 days.

Parameters	Local weather stations			
	Penrith	Observatory park	Sydney airport	Olympic park
Correlation coefficient	0.975	0.981	0.986	0.985
Mean bias error	0.352	0.932	0.429	0.563
Mean absolute error	0.523	0.926	0.432	0.501
Root mean square error	1.023	1.031	1.102	1.361
Index of agreement	0.961	0.954	0.956	0.970
Correlation coefficient	0.972	0.975	0.981	0.982

For determining the UHI intensity, we used both urban and rural weather stations. The magnitude ranged from 3.2–6.3 °C for urban and rural weather stations, respectively. But, we need to reframe the narrative around extreme heat in cities to de-emphasize ‘the concept of UHI’ due to a number of concerns with a focus on urban–rural temperature differences (we can care about absolute conditions not urban–rural differences), and the common confusion between surface versus air temperature UHI (e.g. spatial or temporal difference refers to intensity). This is due to the fact that rural surface temperature and ambient air temperature are mainly sensitive to change in availability of soil moisture, but urban temperatures are still modulated by the dynamics of sensible and storage heat of the urban building environments. Furthermore, assessing UHI severity using surface temperature or ambient air temperature ignores other meteorological factors crucial to how individuals perceive heat, such as complex effects of temperature, humidity and wind speed.

The daily average 2-m ambient air temperature was somewhat overestimated by the model, which might be related to an overestimation of anthropogenic heating over the urban domain. We also assess the effects on local meteorological stations, as they are the ones most touched by the WRF/BEP + BEM scheme’s utility. The well-simulated daytime warming is offset by an equally well-simulated night cooling, resulting in a diurnal range with magnitudes equivalent to observations. In this case, while simulations may capture daytime warming and nighttime cooling well, discrepancies in the diurnal temperature range can arise due to various factors, including local urban influences, cloud cover, heat transfer processes, microscale variations, and data quality. Understanding and improving these aspects can help to enhance the accuracy of simulations and align them more closely with observed diurnal temperature patterns. If the errors in simulating daytime warming and nighttime cooling offset each other, it is possible for the simulated diurnal range to match the observations in terms of magnitude. In this case, the overall temperature range throughout the day could be similar to what is observed, even if the individual components are not accurately represented. The comfort level of various dew points for the stations is > 20 °C, suggesting an uncomfortable situation in the urban building environments. When it comes to analyzing the effects on local meteorological stations, there is no difference. Despite the fact that WRF does not display considerable warm (comfort) bias over urban regions, it properly depicts the 24-h mean diurnal range of dew point temperature. Furthermore, model biases are most likely caused by (a) a lack of proper urban morphological representation, and (b) inaccuracies in model physical schemes, input data used, and locally significant urban biophysical parameters. Despite this, prior studies by Garshasbi et al. (2023a; 2023b)^20,39^ demonstrated that the model is capable of precisely mimicking the actual urban environment of Sydney, including well-simulated evolution of the diurnal cycle of both near-surface and dew point temperature. Thus, the model framework can be utilized to predict regional meteorology and then use the mesoscale WRF model to investigate the local urban warming effects of PVSPs.

## Data Availability

The datasets used and analyzed in this study are available from the corresponding author upon reasonable request.

## References

[1] Sailor DJ, Anand J, King RR (2021). Photovoltaics in the built environment: A critical review. Energy Build..

[2] Barron-Gafford GA, Minor RL, Allen NA, Cronin AD, Brooks AE, Pavao-Zuckerman MA (2016). The Photovoltaic Heat Island Effect: Larger solar power plants increase local temperatures. Sci. Rep..

[3] Taha H (2008). Meso-urban meteorological and photochemical modeling of heat island mitigation. Atmos. Environ..

[4] Taha H (2013). The potential for air-temperature impact from large-scale deployment of solar photovoltaic arrays in urban areas. Sol. Energy.

[5] Ma S, Goldstein M, Pitman AJ, Haghdadi N, MacGill I (2017). Pricing the urban cooling benefits of solar panel deployment in Sydney, Australia. Sci. Rep..

[6] Berardi U, Graham J (2020). Investigation of the impacts of microclimate on PV energy efficiency and outdoor thermal comfort. Sustain. Cities Soc..

[7] Hasan A, Alnoman H, Rashid Y (2016). Impact of integrated photovoltaic-phase change material system on building energy efficiency in hot climate. Energy Build..

[8] Pham JV, Baniassadi A, Brown KE, Heusinger J, Sailor DJ (2019). Comparing photovoltaic and reflective shade surfaces in the urban environment: Effects on surface sensible heat flux and pedestrian thermal comfort. Urban Clim..

[9] Salamanca F, Georgescu M, Mahalov A, Moustaoui M, Martilli A (2016). Citywide impacts of cool roof and rooftop solar photovoltaic deployment on near-surface air temperature and cooling energy demand. Bound.-Layer Meteorol..

[10] Masson V, Bonhomme M, Salagnac JL, Briottet X, Lemonsu A (2014). Solar panels reduce both global warming and urban heat island. Front. Environ. Sci..

[11] Martilli A, Clappier A, Rotach MW (2002). An urban surface exchange parameterisation for mesoscale models. Bound.-Layer Meteorol..

[12] Salamanca F, Krpo A, Martilli A, Clappier A (2010). A new building energy model coupled with an urban canopy parameterization for urban climate simulations—Part I. Formulation, verification, and sensitivity analysis of the model. Theor. Appl. Climatol..

[13] Broadbent AM, Krayenhoff ES, Georgescu M, Sailor DJ (2019). The observed effects of utility-scale photovoltaics on near-surface air temperature and energy balance. J. Appl. Meteorol. Climatol..

[14] Oh, J., TamizhMani, G. & Palomino, E. Temperatures of building applied photovoltaic (BAPV) modules: Air gap effects. In *Reliability of Photovoltaic Cells, Modules, Components, and Systems III*. vol. 7773, 33–43 (SPIE, 2010).

[15] Liu S, Yan Y, Zhang Z, Bai J (2019). Effect of distributed photovoltaic power station on cooling load induced by roof for sunny day in summer. Therm. Sci. Eng. Progress.

[16] Wang D, Qi T, Liu Y, Wang Y, Fan J, Wang Y, Du H (2020). A method for evaluating both shading and power generation effects of rooftop solar PV panels for different climate zones of China. Sol. Energy.

[17] Kapsalis V, Karamanis D (2015). On the effect of roof added photovoltaics on building's energy demand. Energy Build..

[18] Brown KE, Baniassadi A, Pham JV, Sailor DJ, Phelan PE (2020). Effects of rooftop photovoltaics on building cooling demand and sensible heat flux into the environment for an installation on a white roof. J. Eng. Sustain. Build. Cities.

[19] Zonato A, Martilli A, Gutierrez E, Chen F, He C, Barlage M, Giovannini L (2021). Exploring the effects of rooftop mitigation strategies on urban temperatures and energy consumption. J. Geophys. Res. Atmos..

[20] Garshasbi, S., Khan, A. & Santamouris, M. On the cooling energy penalty of urban photovoltaics: A case study in Sydney, Australia. *Energy Build*. 113259 (2023b).

[21] Quinting JF, Reeder MJ (2017). Southeastern Australian heat waves from a trajectory viewpoint. Mon. Weather Rev..

[22] Chen F, Kusaka H, Bornstein R, Ching J, Grimmond CSB, Grossman-Clarke S, Zhang C (2011). The integrated WRF/urban modelling system: Development, evaluation, and applications to urban environmental problems. Int. J. Climatol..

[23] Santamouris M, Haddad S, Fiorito F, Osmond P, Ding L, Prasad D, Wang R (2017). Urban heat island and overheating characteristics in Sydney, Australia. An analysis of multiyear measurements. Sustainability.

[24] Cortes A, Murashita Y, Matsuo T, Kondo A, Shimadera H, Inoue Y (2015). Numerical evaluation of the effect of photovoltaic cell installation on urban thermal environment. Sustain. Cities Soc..

[25] Scherba A, Sailor DJ, Rosenstiel TN, Wamser CC (2011). Modeling impacts of roof reflectivity, integrated photovoltaic panels and green roof systems on sensible heat flux into the urban environment. Build. Environ..

[26] Jones AD, Underwood CP (2002). A modelling method for building-integrated photovoltaic power supply. Build. Serv. Eng. Res. Technol..

[27] NREL, N. Best research-cell efficiencies chart (2019).

[28] DoE, U. S. Energyplus engineering reference. The reference to energyplus calculations, 1 (2010).

[29] Skamarock WC, Klemp JB (2008). A time-split nonhydrostatic atmospheric model for weather research and forecasting applications. J. Comput. Phys..

[30] Salamanca F, Martilli A, Tewari M, Chen F (2011). A study of the urban boundary layer using different urban parameterizations and high-resolution urban canopy parameters with WRF. J. Appl. Meteorol. Climatol..

[31] Salamanca F, Georgescu M, Mahalov A, Moustaoui M, Wang M, Svoma BM (2013). Assessing summertime urban air conditioning consumption in a semiarid environment. Environ. Res. Lett..

[32] Chen F, Dudhia J (2001). Coupling an advanced land surface–hydrology model with the Penn State–NCAR MM5 modeling system. Part I: Model implementation and sensitivity. Mon. Weather Rev..

[33] Ek, M. B., Mitchell, K. E., Lin, Y., Rogers, E., Grunmann, P., Koren, V. & Tarpley, J. D. Implementation of Noah land surface model advances in the National Centers for Environmental Prediction operational mesoscale Eta model. *J. Geophys. Res. Atmos*. **108**(D22) (2003).

[34] Mellor GL, Yamada T (1974). A hierarchy of turbulence closure models for planetary boundary layers. J. Atmos. Sci..

[35] Dudhia J (1989). Numerical study of convection observed during the winter monsoon experiment using a mesoscale two-dimensional model. J. Atmos. Sci..

[36] Mlawer EJ, Taubman SJ, Brown PD, Iacono MJ, Clough SA (1997). Radiative transfer for inhomogeneous atmospheres: RRTM, a validated correlated-k model for the longwave. J. Geophys. Res. Atmos..

[37] Bougeault P, Lacarrere P (1989). Parameterization of orography-induced turbulence in a mesobeta–scale model. Mon. Weather Rev..

[38] Kain JS (2004). The Kain-Fritsch convective parameterization: An update. J. Appl. Meteorol..

[39] Garshasbi S, Feng J, Paolini R, Duverge JJ, Bartesaghi-Koc C, Arasteh S, Santamouris M (2023). On the energy impact of cool roofs in Australia. Energy Build..

